# Predicting room temperature phosphorescence of new organic molecules by combining physical models and data-driven methods

**DOI:** 10.1039/d6sc06125g

**Published:** 2026-09-08

**Authors:** Xia Wu, Alessandro Troisi

**Affiliations:** a Department of Chemistry, University of Liverpool Liverpool L69 3BX UK xia.wu@liverpool.ac.uk A.Troisi@liverpool.ac.uk

## Abstract

The rational discovery of new RTP emitters remains challenging because phosphorescence depends on multiple competing processes, including intersystem crossing, nonradiative decay, and competition with fluorescence. While several mechanistic hypotheses have been proposed, like enhanced spin–orbit coupling, reduced energy gap between singlet and triplet states, and favourable electronic transition properties, the extent to which these factors can quantitatively predict RTP behaviour remains unclear. In this work, we have collected a dataset of 218 metal-free organic RTP emitters from the literature. We first explored physical approaches to predict phosphorescence yield using descriptors derived from established mechanisms and hypotheses, such as favourable spin–orbit coupling, reorganization energy, and oscillator strength. Although these quantities show statistically meaningful correlations with RTP behaviour, the physical models alone are insufficient to construct a robust quantitative predictive tool. We therefore developed a data-driven machine learning (ML) model based on the Random Forest algorithm, combining these physically motivated descriptors with additional topological descriptors derived from the molecular structure. After feature selection, the optimized models achieved prediction accuracies of 0.774 and 0.820 identifying long-lifetime (lifetime > 90 ms) and long-wavelength (RTP emission > 525 nm) RTP materials, respectively. With the boundary from the physical process and ML analysis, 65 candidates are identified from 48 168 molecules with predicted probabilities greater than 0.75 for both long-lifetime and long-wavelength properties, providing a focused set of promising materials for further experimental validation.

## Abstract 2

We collected 218 metal-free organic RTP emitters and developed a Random Forest model combining physical/topological descriptors. It identified long-lifetime and long-wavelength RTP materials with accuracies of 0.774 and 0.820, yielding 65 candidates.

## Graphical abstract

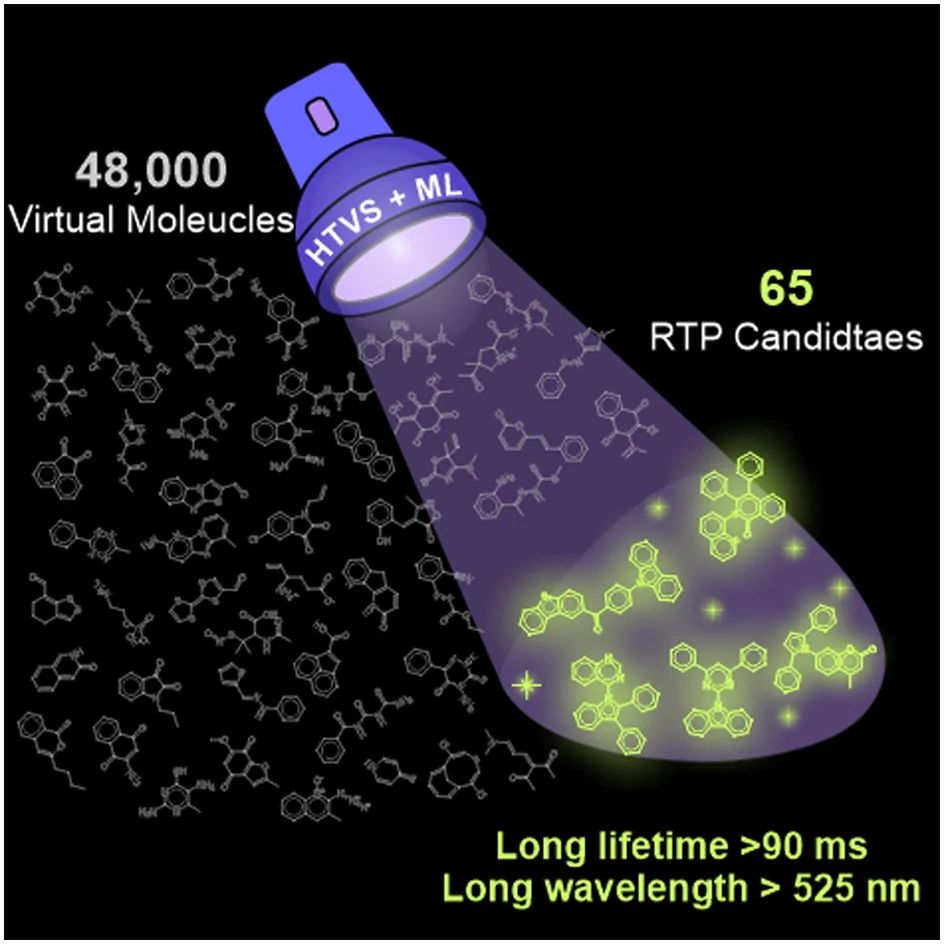

## Introduction

Room-temperature phosphorescence (RTP) is an optical phenomenon in which organic materials emit light *via* triplet exciton decay after removal of the excitation source, persisting well beyond typical fluorescence lifetimes at room temperature.^1^ This arises from spin-forbidden T_1_ → S_0_ transitions facilitated by intersystem crossing (ISC), where intermolecular interactions (*e.g.* hydrogen bonding,^2,3^ halogen bonding,^4–6^ and π–π stacking^7,8^) suppress non-radiative decay and stabilize triplet excitons.^9,10^ The resulting long emission lifetimes and large Stokes shifts have made RTP materials attractive for a wide range of applications, including time-gated bioimaging,^11,12^ anti-counterfeiting,^13,14^ organic light-emitting diodes (OLEDs),^15,16^ and optical sensing,^17,18^ making them promising candidates for next-generation luminescent functional materials.

Despite these promising prospects, the rational design and prediction of RTP behaviour remain challenging. Traditionally, molecular design strategies have focused primarily on the single molecule level.^19,20^ Within this framework, descriptors such as singlet–triplet energy gaps (Δ*E*_ST_), spin–orbit coupling (SOC) matrix elements, excited state energies, and charge-transfer character have been widely employed to assess RTP property.^21–23^ However, reliable calculations remain difficult when multiple descriptors need to be considered. For example, SOC is often weak and challenging to calculate accurately in large amount of organic systems,^24^ and RTP performance depends on a complex balance between intersystem crossing, triplet-state decay, and competing non-radiative pathways, many of which remain difficult to model quantitatively with good agreement with experiment, not to mention to a dataset with large amount molecules.^25–27^ That is why some molecules with similarly favourable electronic characteristics can exhibit different RTP behaviour experimentally.^28,29^

More fundamentally, the single-molecule perspective overlooks the fact that RTP is mainly observed in the solid state.^30,31^ Several factors influence the triplet excitons' survival time, most notably packing mode^32–35^ and hydrogen bonding,^36–39^ alongside molecular conformation,^40–42^ and specific intermolecular interaction.^43–45^ This is a second reason that compounds with related structures can exhibit completely different phosphorescence behavior.^4,46,47^ Although a few studies have attempted to predict RTP property using QM/MM approaches, the results are only for single target molecules with different packing modes.^48,49^ Thus, the discovery of new RTP candidates has largely relied on inefficient trial-and-error synthesis guided by empirical intuition.

High-throughput virtual screening (HTVS) has emerged as a powerful approach for rapidly exploring vast chemical spaces at a same level of the experimental cost. It has been successfully deployed to identify luminescent materials such as emitters with inverted singlet-triplet energy gaps,^50–54^ singlet fission (SF) chromophores,^55–58^ and thermally activated delayed fluorescence (TADF) materials,^59–63^ demonstrating its potential to accelerate functional materials discovery.^64^ However, applying only HTVS to screen RTP is still inefficient. The lack of high-quality experimental RTP datasets limits the validation of descriptors; in parallel, the low computational accuracy of some photophysical parameters and the difficulty of accounting for crystal-state effects make the screening results unreliable. The inclusion of machine learning (ML) methods can accelerate the screening and offer the potential to uncover structure–property relationships that are not easily captured by physics-based descriptors alone. Despite this potential, successful examples of combining HTVS with ML (HTVS-ML) to screen organic RTP materials remain very rare. One such example, reported in ref. 65, demonstrated this approach for cocrystal RTP materials.^65^ In another study, Ma *et al.* constructed a database of ∼20 000 candidates by combining 25 ISC-active fragments with 27 phosphorescent groups showing long lifetimes at 77 K, then built a workflow that combines ground-state orbital descriptors (πFMOs), machine learning, excited-state calculations, and experimental validation to predict long-lived RTP molecules.^66^ Still, most candidates in their database are hypothetical combinations with uncertain synthetic feasibility, and the reliance on ground-state descriptors may not fully capture the excited-state processes that govern RTP.

In this work, we established a dataset of 218 pure organic RTP materials with experimental photophysical properties. Then, key physical descriptors are calculated, including the energies of the first vertical singlet and triplet excited states (*E*_S_1__ and *E*_T_1__), oscillator strength (*f*), spin–orbit coupling (SOC), and triplet excited state reorganization energies (*λ*_T_1__). Based on a Cambridge Structural Database (CSD) candidates pool^67^ and following analysis of the ML model, we identified several RTP candidates with predicted lifetimes exceeding 90 ms and emission wavelengths greater than 525 nm.

## Methods

### Key factors influencing phosphorescence quantum yield in RTP

The phosphorescence quantum yield *Φ*_p_ for RTP can be expressed as:^1^1
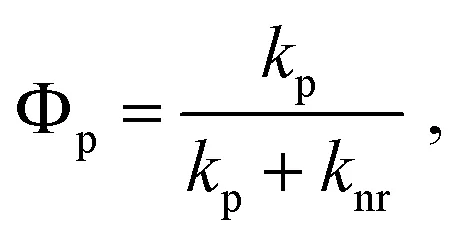
where *k*_p_ is the radiative decay rate, *k*_nr_ is the nonradiative decay rate during the phosphorescence process.

According to first-order perturbation theory, *k*_p_ can be approximately expressed by:^68–70^2
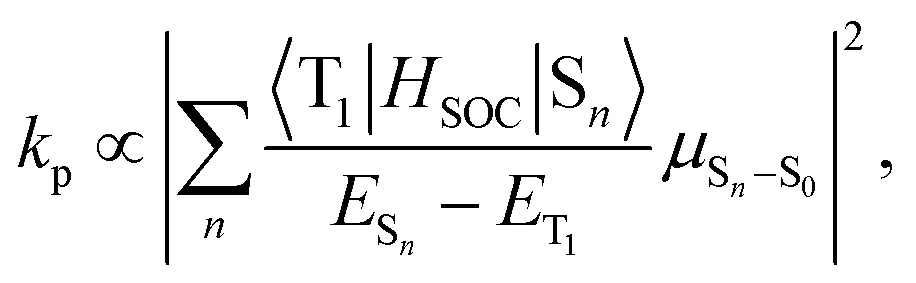
where *µ*_S_*n*_−S_0__ is the transition dipole moment between S_*n*_ and S_0_ state, T_1_|*H*_SOC_|S_*n*_ is the SOC between S_*n*_ and T_1_, and *E*_S_*n*__ − *E*_T_1__ is the energy difference between S_*n*_ and T_1_ states.

Moreover, the oscillator strength can be calculated from a quantum mechanical expression as^71,72^3
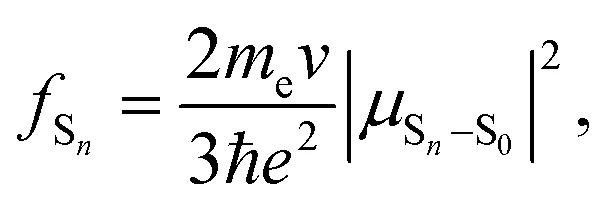
where *v* is the frequency of the transition, *ℏ* is the reduced Planck constant. We can get4
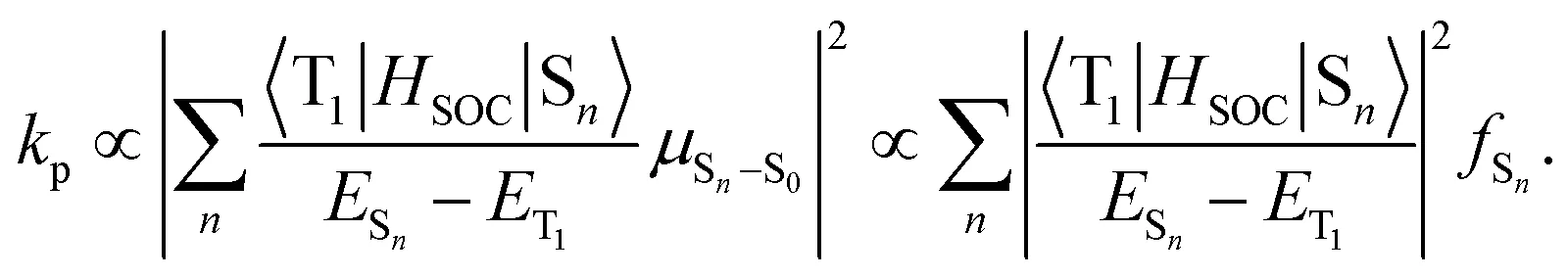


The energy gap law relates *k*_nr_ of a transition to the energy gap between the states involved. In its simplest form for *k*_nr_ from T_1_ to S_0_, it may be written as:^73–75^

5 *k*_nr_ ∝ 〈T_1_|*H*_SOC_|S_0_〉^2^exp(−*β*Δ*E*),

where T_1_|*H*_SOC_|S_0_ is the SOC between T_1_ to S_0_, Δ*E* is the gap between T_1_ to S_0_, and *β* is a term that can be expressed in terms of molecular parameters, which correlates negatively with the reorganization energy of the T_1_ (*λ*_T_1__). In this work, *λ*_T_1__ is defined as the energy gap between the vertical triplet excitation from the equilibrium S_0_ geometry and the relaxed minimum of the adiabatic T_1_ state.

Accordingly, improving phosphorescence quantum yield requires strengthening the SOC between S_*n*_ and T_1_ (SOC_S_*n*_T_1__) and increasing the oscillator strength of the S_*n*_ (*f*_S_*n*__), while reducing the energy gap between S_*n*_ and T_1_ (Δ*E*_S_*n*_−T_1__). For getting slow non-radiative decay rate, larger energy gap between T_1_ and S_0_ (Δ*E*_T_1_−S_0__) and smaller SOC_T_1_S_0__, as well as small *λ*_T_1__. If nonradiative decay from T_1_ to S_0_ dominates at room temperature, it outcompetes phosphorescent emission, and RTP is not observed.

### RTP database

To establish a reliable foundation for screening, we collected a dataset of 218 pure organic RTP molecules with phosphorescence lifetimes exceeding 0.1 ms from the literature (shared on GitHub by https://github.com/XiaWu-Liv/RTP-databases/). Each entry is traceable to its originating publication *via* DOI, ensuring full transparency and reusability. The dataset includes experimental properties such as phosphorescence lifetime (*τ*_p_), RTP emission wavelength, phosphorescence quantum yield (*Φ*_p_), radiative decay rate (*k*_p_), intersystem crossing rate (*k*_ISC_), and nonradiative decay rate (*k*_nr_). Among these properties, phosphorescence lifetime and RTP emission wavelength were available for all molecules (distributions are in Fig. S1), whereas the remaining properties were reported only for a subset of the dataset. A selection of molecules with a simplified chemical classification is given in Fig. 1a. We recognized that the dataset includes measurements obtained under diverse experimental conditions from different research groups, which may contribute noise to the training data. Furthermore, RTP behaviour for the same molecule can vary depending on crystal packing. To address this, when multiple literature values were available for a given molecule, the best performing result was selected for inclusion in the dataset.

**Fig. 1:**
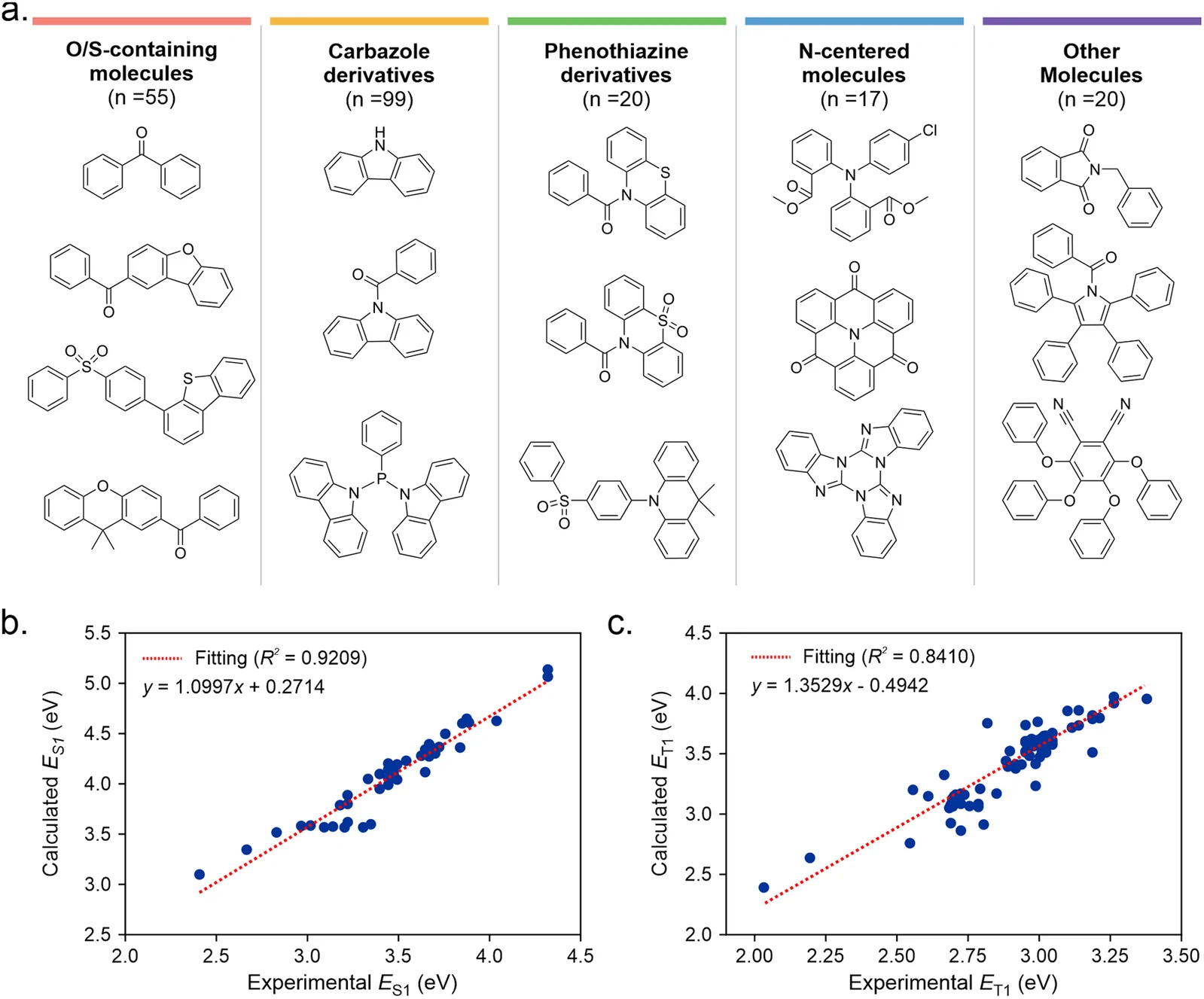
(a) Representative examples from the RTP dataset, consisting of 218 materials classified into five categories. Calibration of the calculated and experimental energies for (b) the first singlet excited state (S_1_) and (c) the first triplet excited state (T_1_). (b) Includes 47 experimental absorption data points, while (c) includes 118 experimental phosphorescence data points measured in solvents at 77 K.

Also, a set of photophysical descriptors was computed, including *E*_S_1__, *E*_T_1__, Δ*E*_ST_, *f*, the frontier molecular orbital energies (HOMO and LUMO), SOC constants, and *λ*_T_1__. The computed *E*_S_1__ and *E*_T_1__ show good agreement with experimental data, yielding *R*^2^ values of 0.92 and 0.84, respectively, validating the reliability of the computational protocol adopted for downstream screening (Fig. 1b and c).

### Computational methods

All density functional theory (DFT) and time-dependent DFT (TDDFT) calculations were carried out using the Gaussian 16A suite of programs,^76^ except for SOC constants. For vertical excited state energies, geometry optimizations were carried out at the BLYP35/3-21G(d) level and corresponding TDDFT calculations were utilizing the M06-2X with def2-TZVP,^50,77^ which is aligned with the level used in the large dataset later. This methodology achieves high agreement with experimental data at relatively low computational cost, making it well-suited for large datasets. The molecular excitation properties were also investigated by the hole–electron analysis using Multiwfn 3.8.^78,79^ The *λ*_T_1__ calculations were performed at the M06-2X/def2-TZVP for whole processes to ensure methodological consistency. For RTP dataset, *λ*_T_1__ was obtained for 205 molecules, while calculations for the remaining molecules failed to converge in the adiabatic T_1_ excited state.

SOC constants are obtained by ORCA with M06-2X/def2-TZVP.^80^ For SOC values from S_1_ to T_1_, we use the geometry of the optimized ground state S_0_, and SOC values from T_1_ to S_0_ is based on the optimized geometries of T_1_ state.

### ML model

To predict the photophysical properties of RTP, a Random Forest classifier was trained using 24 molecular descriptors as input features. The dataset of 218 RTP molecules consisted exclusively of experimentally confirmed RTP materials and therefore lacked negative examples (non-RTP molecules). Furthermore, the relatively low consistency of data was reported across different laboratories. Therefore, classification into RTP *versus* non-RTP, or precise regression of lifetime and wavelength values, was not considered appropriate. Instead, the prediction task was reformulated as a binary classification problem by categorical labels: RTP lifetime into short- and long-lived categories and emission wavelength into short- and long-wavelength categories (several selected thresholds are tested and the one with the highest accuracy and F1 was picked). Model performance was then evaluated using classification metrics derived from the confusion matrix, with true positives (TP), true negatives (TN), false positives (FP), and false negatives (FN) calculated from the predicted and actual class labels.^81–83^

Model robustness was evaluated using *k*-fold cross-validation with *k* = 10, ensuring that the dataset was partitioned into ten subsets, with each subset serving once as the validation fold while the remaining nine were used for training. A fixed “random_state = 42” was applied throughout to control the randomness of the data splitting and the Random Forest's internal bootstrap sampling, ensuring reproducibility of the results across runs.

Hyperparameter optimization was performed through a grid search over two key Random Forest parameters: the maximum tree depth (max_depth), sampled over the range 1 to 20, and the number of estimators (*n*_estimators), sampled over the range 1 to 200. For each combination of these parameters, the model was trained and validated within the 10-fold cross-validation scheme, and the configuration yielding the best average performance across folds was selected as the final model.

Model performance was quantified using two complementary metrics computed from the confusion matrix. Accuracy measures the overall proportion of correctly classified instances, defined as6
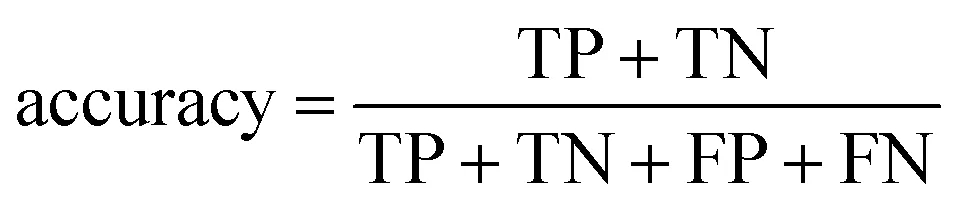


The F1 score was additionally calculated to account for the balance between precision and recall, it is defined as7
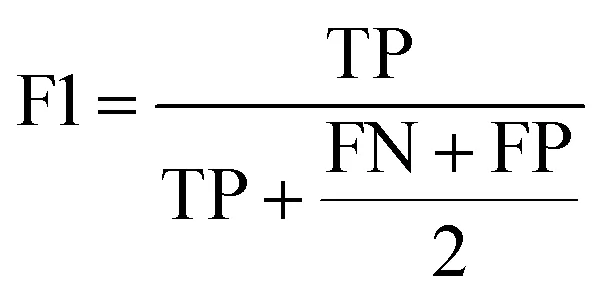


Together, these metrics provided a comprehensive assessment of the ability of Random Forest model to accurately classify the region of RTP lifetime and emission wavelength based on the 24 molecular descriptors.^84,85^ The performance of individual molecules was further evaluated using the predicted class probabilities, which were calculated as the mean class probabilities predicted by all decision trees in the Random Forest. These probabilities were then used to rank candidate RTP materials during the screening process.

Specifically, for the threshold of the binary classification problem, a 90 ms threshold was used to classify RTP lifetime as short- or long-lived, and a 525 nm threshold was used to classify emission wavelength as short- or long-wavelength (the rationale for these values is discussed later). These thresholds enabled the Random Forest model to distinguish RTP materials with more desirable photophysical properties for virtual screening.

## Results and discussion

### From descriptors analysis to machine learning model

First, we investigate the descriptors discussed above to assess whether any exhibit a clear threshold that could be used to screen RTP candidates. To this end, we examined the distributions of the *E*_S_1__, *E*_T_1__, Δ*E*_S_1_T_1__, *f*_S_1__, SOC_S_1_T_1__, SOC_T_1_S_0__, and *λ*_T_1__ across the RTP dataset (Fig. 2). *E*_S_1__ is located between 3.0–5.4 eV while *E*_T_1__ falls between 2.2–4.2 eV. The Δ*E*_S_1_T_1__ and *λ*_T_1__ show similarly smooth, single-peaked distributions, with Δ*E*_S_1_T_1__ peaking near 0.7 eV and *λ*_T_1__ centered near 0.3–0.5 eV. The distributions of *f*_S_1__ and both SOC terms are more irregular and broadly dispersed. Overall, none of these descriptors showed a sufficiently sharp or well-defined threshold suitable for HTVS. Nevertheless, *E*_S_1__ and *E*_T_1__ still offer relatively narrow energy windows that can be applied in initial screening steps to reduce the candidate's pool. Even though only a few molecules emit in the visible based on *E*_T_1__ value, the deeper issue is that most triplet states are being lost through non-radiative decay and increasing the S_0_–T_1_ gap can actually benefit RTP by suppressing these non-radiative pathways.

**Fig. 2:**
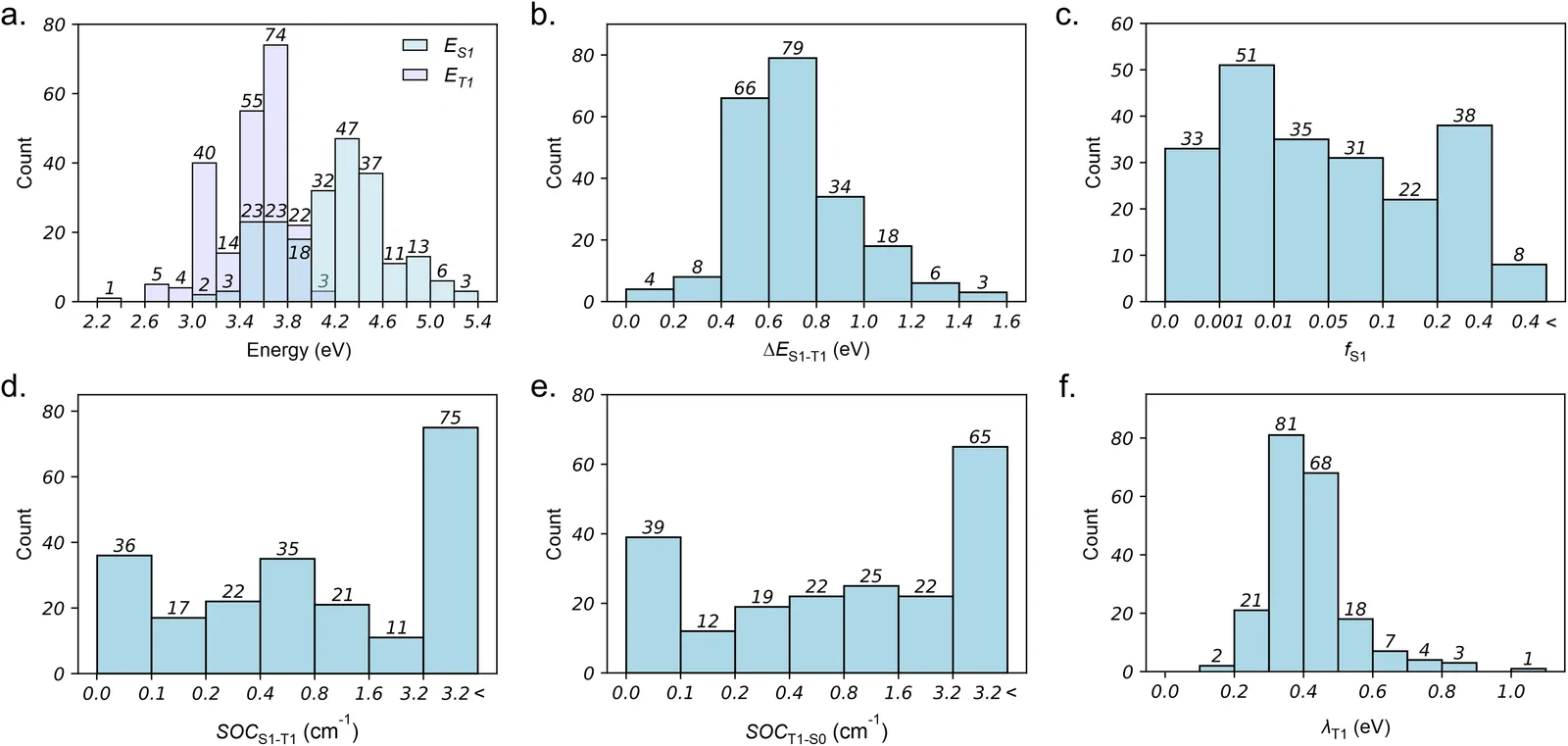
The distribution of (a) *E*_S_1__ and *E*_T_1__, (b) Δ*E*_S_1_T_1__, (c) *f*_S1_, (d) SOC_S_1_T_1__, (e) SOC_T_1_S_0__, and (f) *λ*_T_1__ for the RTP dataset. Since one molecule in the dataset contains an iodine(I) atom, panels (d) and (e) include 217 constants.

The same situation is observed for the descriptors based on the phosphorescence rate equation (Fig. 3): regardless of the variant of the descriptor used, no reliable threshold can be established. This explains why conventional HTVS screening strategies could not be applied directly to the discovery of RTP materials. More complex correlations could be derived using globally all data and applying ML methods.

**Fig. 3:**
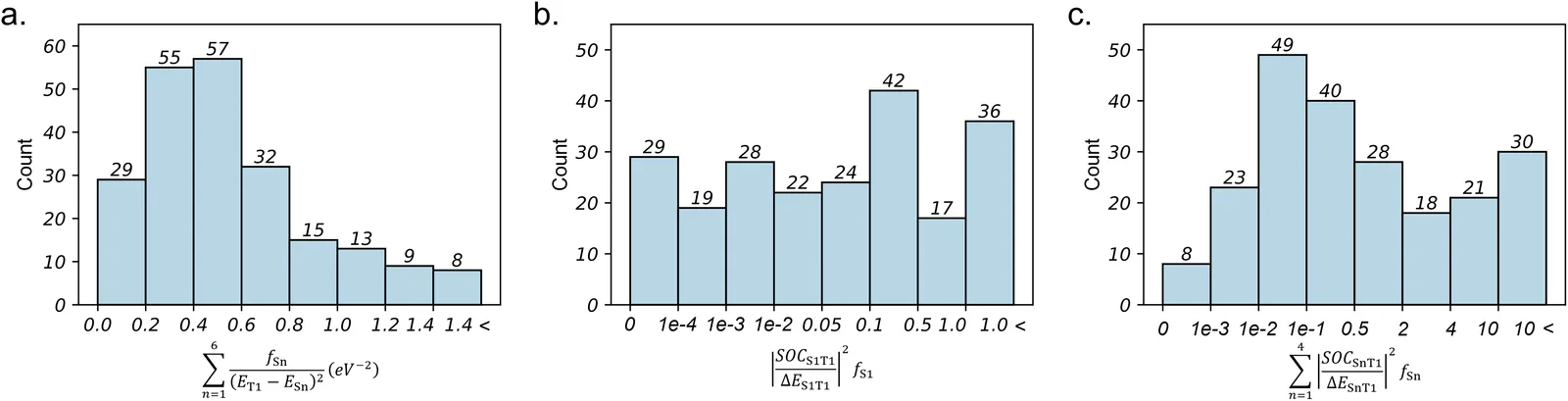
The distribution of a (a) 
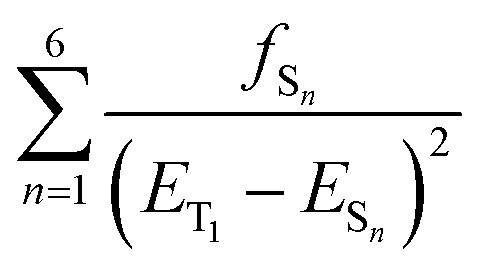
, (b) 
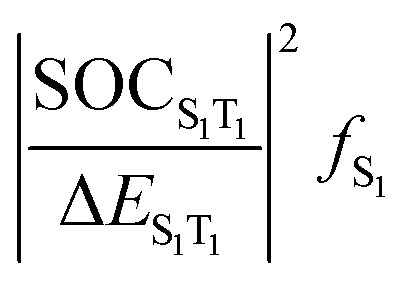
, (c) 
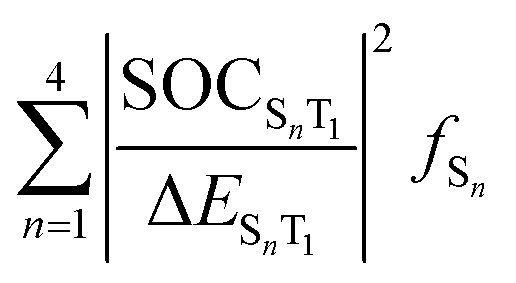
.

Since the lifetime and the RTP wavelength are two target properties available for every candidate in the dataset, we investigated their relationship with the other descriptors. In Fig. 4, we present a Pearson heatmap of 24 descriptors for the RTP dataset against emission lifetime and RTP wavelength, covering electronic structure parameters (*e.g.*, HOMO, LUMO, *E*_S_1__, *E*_T_1__, Δ*E*_S_1_T_1__), excited-state spatial separation indices (*D* index and *t* index for S_1_ and T_1_).^78^ Details of all descriptors are in Table S1. In addition to these quantum-chemical descriptors, a set of molecular and topological descriptors (*e.g.*, molecular weight, aromaticity, H-bonding capacity, TPSA, and WLOGP) computed from RDKit are added,^86–88^ which can be directly derived from SMILES strings at negligible computational cost. This inclusion allows us to evaluate both inter-descriptor redundancy and the relevance of each descriptor to the two target properties prior to model training. However, the correlation analysis indicates that none of the descriptors exhibit strong linear correlations with either lifetime or RTP wavelength.

**Fig. 4:**
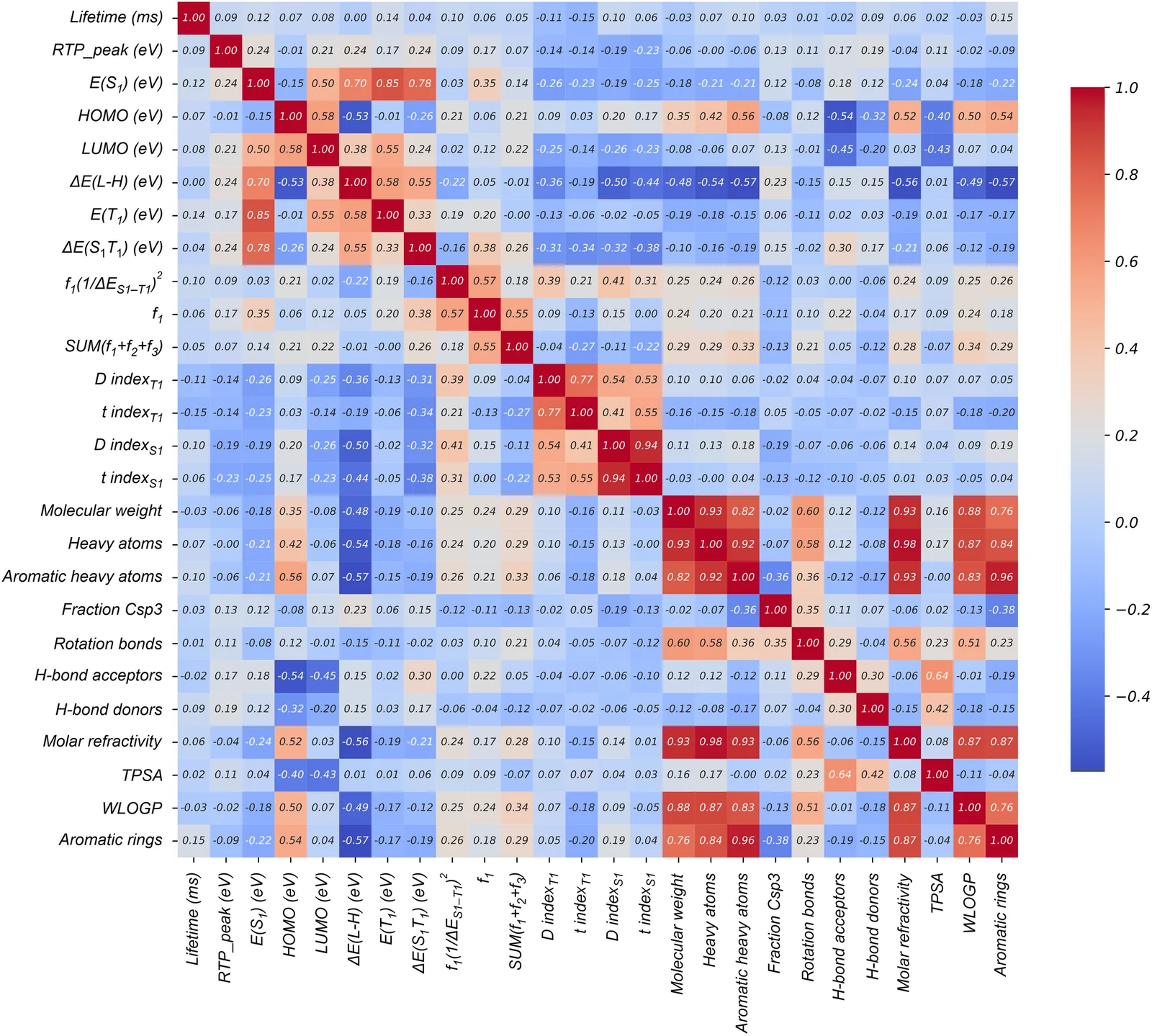
Pearson heatmap of the parameters using in the ML model.

Given this level of correlation, we formulated the prediction task as a binary classification problem: samples were labelled based on whether their measured lifetime fell above or below a defined threshold value. Three thresholds were evaluated—80 ms, 90 ms, and 100 ms—to assess the sensitivity of model performance to the choice of decision boundary (details of method are in Table. S2). Hyperparameter optimization was performed independently for each threshold, with max_depth explored in the range 1–21 and *n*_estiamtors in the range 1–201. The optimal classification performance was obtained using a lifetime threshold of 90 ms, resulting in an accuracy of 0.774 and an F1 score of 0.846. The corresponding confusion matrix showed 135 correctly identified long-lifetime emitters (TP) and 33 correctly identified short-lifetime emitters (TN), together with 14 FN and 35 FP cases. The 80 ms threshold produced a comparable F1 score (0.844) but slightly lower accuracy (0.770), while the 100 ms threshold performed least well (accuracy: 0.756, F1: 0.834). The performance at 90 ms suggests this threshold best separates the underlying material property distributions captured by the 24 input descriptors and was therefore selected as the operational decision boundary for the final screening model.

Analogous to the lifetime classification, the RTP emission wavelength was also treated as a binary classification target. Samples were labelled according to whether their peak emission wavelength fell above or below a defined threshold, distinguishing short-wavelength (blue/cyan) emitters from longer-wavelength emitters. This distinction is practically motivated, as green-to-NIR emission is strongly preferred in bioimaging, anti-counterfeiting, and optoelectronic devices, where deeper tissue penetration and higher visual contrast are desirable.^89–91^ Three threshold values were evaluated: 475 nm, 500 nm, and 525 nm (details of the method are in Table S3). While the 475 nm threshold achieved the highest raw accuracy (0.880) and F1 score (0.934), its highly imbalanced confusion matrix (only 9 TN *vs.* 182 TP) indicates that the dataset is heavily skewed toward longer-wavelength emitters at this cutoff, limiting its discriminative utility. The 525 nm threshold was then selected as the operational boundary, yielding an accuracy of 0.820 and an F1 score of 0.859, with a more balanced class distribution (59 TN, 119 TP).

To validate the selected probability thresholds for a lifetime over 90 ms and a wavelength over 525 nm, the corresponding ML models were applied retrospectively to the RTP dataset (probabilities were calculated as the mean class probabilities predicted by all decision trees in the Random Forest). At a probability threshold of 0.65, the lifetime model correctly identifies 98.7% (147/149) of RTP materials, while the wavelength model identifies 97.8% (132/135). At the stricter threshold of 0.75, identification rates decrease to 89.3% (133/149) for the lifetime model and 90.4% (122/135) for the wavelength model. Although a higher probability threshold would provide more conservative predictions, it could also exclude a considerable number of potentially promising RTP candidates. Therefore, during the ML-based virtual screening stage, only molecules with probabilities above 0.75 for both the lifetime and wavelength model were retained in this study. The full candidate list is also available on GitHub for molecules with probabilities above 0.65, with detailed probabilities for a lifetime over 90 ms and an emission wavelength over 525 nm, allowing researchers to adjust the threshold according to their specific requirements.

### Virtual screening

The virtual screening pipeline proceeded through a series of sequential filtering stages, as illustrated in Fig. 5. The starting pool consisted of 48 182 molecules with a low HOMO–LUMO gap, extracted from the CSD database,^67^ and for which *E*_S_1__ and *E*_T_1__ have been computed at the M06-2X/def2-SVP level of theory. This dataset is particularly convenient for a possible follow-up version because synthetic routes for the candidates are known alongside the crystalline arrangement. In the first filtering stage, rule-based physicochemical descriptors derived from the 218 experimentally validated RTP molecules were applied as structural constraints, including: number of heavy atoms ≥12, aromatic heavy atoms ≥16, fraction of sp^3^ carbon (Fraction Csp^3^) ≤0.47, molar refractivity (MR) >40, topological polar surface area (TPSA) in the range 0–100 Å^2^, and the presence of at least one aromatic ring. Application of these filters reduced the candidate pool to 33 962 molecules. A second stage of filtering was performed using excited-state energy criteria of 3.51 eV < *E*_S_1__ < 5.02 eV and 2.986 eV < *E*_T_1__ < 3.923 eV. The energy boundaries were defined by retaining the central 90% of the *E*_S_1__ and *E*_T_1__ distributions for the 218 RTP molecules, thereby focusing on the most statistically concentrated region (Fig. S2). The same level of theory with the CSD dataset is used to calculate these properties for the 218 RTP molecules, ensuring methodological consistency. After this filtering step, the candidate pool was narrowed to 4217 molecules, for which all 24 molecular descriptors were then calculated at the M06-2X/def2-TZVP level. The trained Random Forest classifiers were then used to evaluate the full set of 4217 candidates (this dataset is provided on the GitHub for rapid reproducibility). Candidates were then required to satisfy both filters simultaneously, corresponding to a predicted lifetime greater than 90 ms and an RTP emission wavelength longer than 525 nm, each with a probability above 0.75. Applying these combined criteria reduced the candidate pool to 65 molecules for subsequent analysis.

**Fig. 5:**
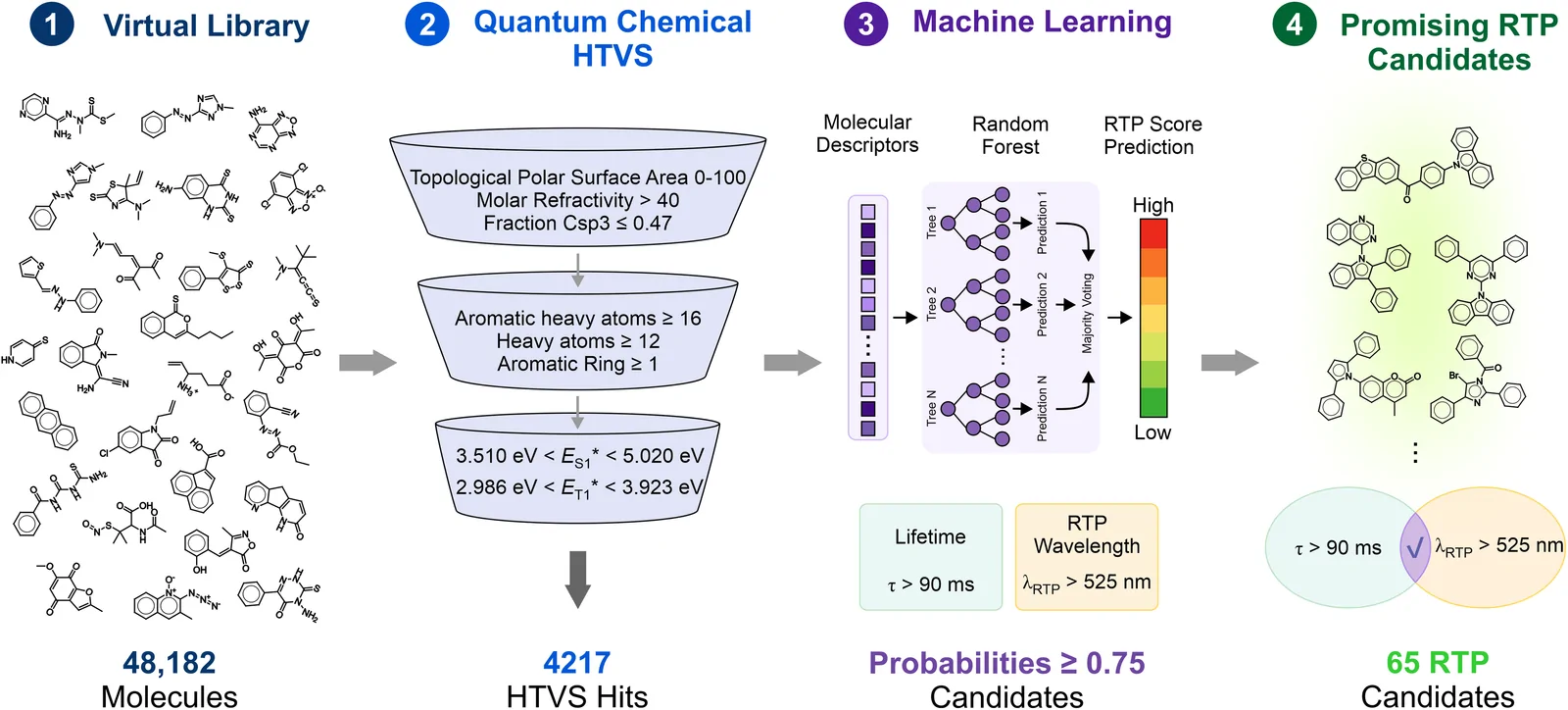
Screening process of RTP materials.

Selected molecular structures from the 65 candidates are listed in Fig. 6, while all 65 molecules are reported in Fig. S3–S5. Notably, three well-known RTP materials are also found among the top-ranked candidates (Fig. S3), supporting the reliability of our model's predictions. Some candidates are structurally similar to known RTP materials or contain highly efficient RTP-active substituents such as carbazole, dibenzofuran, and isoindoline-1,3-dione (Fig. S4). Surprisingly, some candidates have entirely novel structures with respect to the starting literature dataset (Fig. 6 and S5).

**Fig. 6:**
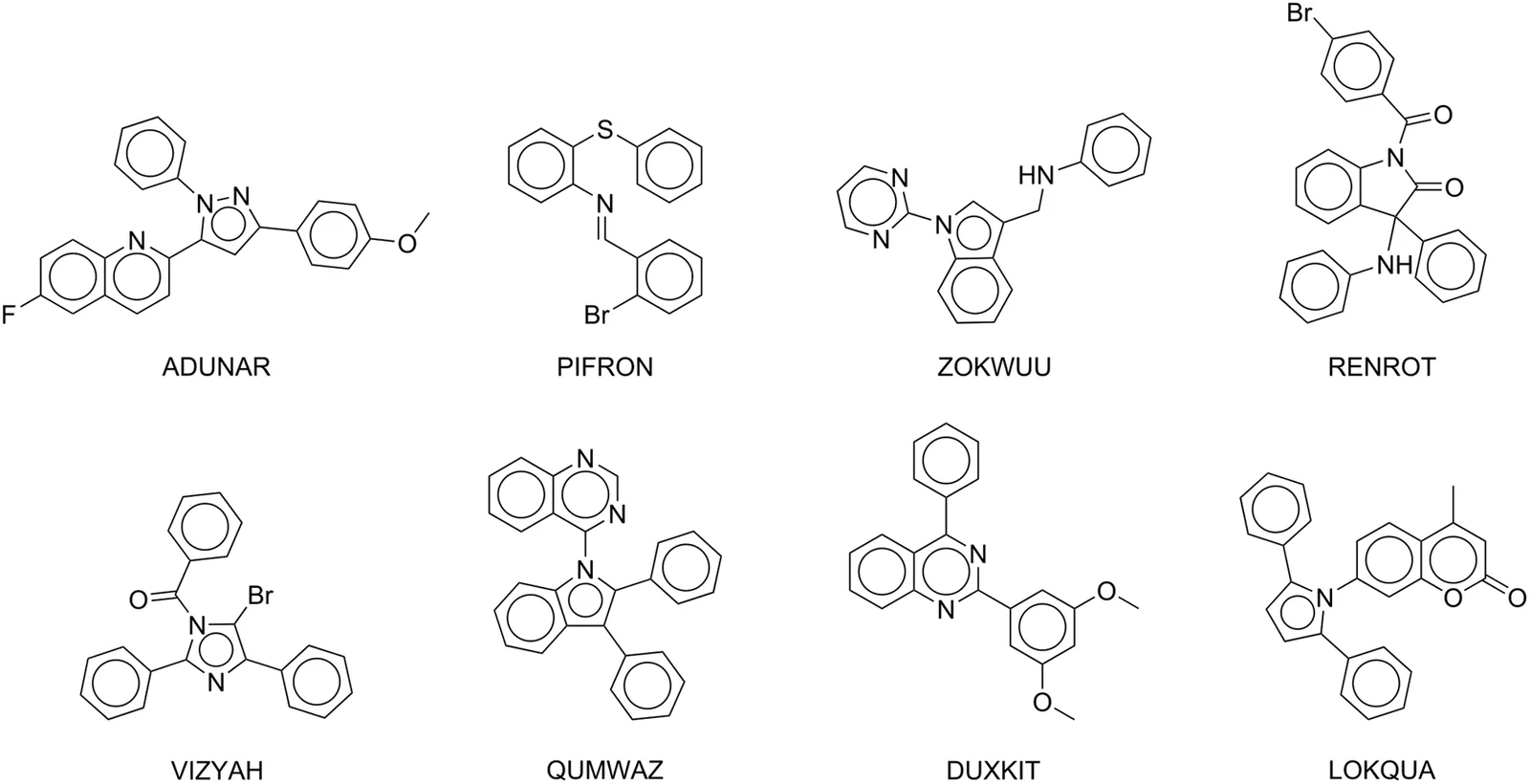
A part of molecular structures of promising RTP candidates featuring novel structural motifs (from 65 candidates, all other candidates are shown in Fig. S5).

To evaluate the excited state photophysical data of representative candidates, a set of descriptors relevant to the RTP mechanism are listed in Table 1. All candidates exhibit acceptable Δ*E*_S_1_T_1__ combined with non-negligible SOC_S_1_T_1__ values, consistent with efficient intersystem crossing. For example, molecule RENROT has a Δ*E*_S_1_T_1__ of 0.62 eV with a SOC_S_1_T_1__ of 5.69 cm^−1^. The relatively low but non-zero oscillator strengths (0 < *f*_S_1__ < 0.05) further support a predominantly n → π* character for the S_1_ states in these systems. *E*_T_1__ calculated at the adiabatic geometries 
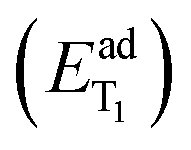
 ensures the RTP emission wavelength falls within the green-NIR region. Small *λ*_T_1__ indicate minimal reorganization upon relaxation to the T_1_ state, which can suppress non-radiative decay; the correspondingly small SOC_T_1_S_0__ values further support this conclusion.

**Table 1:** Part of descriptors for the RTP candidates in Fig. 6

CSD ID	*E*_S_1__(eV)	*f*_S_1__	*E*_T_1__ (eV)	Δ*E*_S_1_T_1__ (eV)	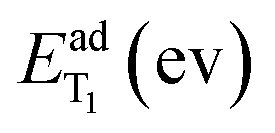	*λ*_T_1__(eV)	SOC_S_1_T_1__ (cm^−^^1^)	SOC_T_1_S_0__ (cm^−1^)
ADUNAR	3.9194	0.0400	3.0011	0.9183	2.1694	0.4800	1.2654	0.9852
PIFRON	3.6718	0.1849	2.9679	0.7039	/a	/a	6.9622	/a
ZOKWUU	4.3703	0.0030	3.5737	0.7966	2.3886	0.5804	1.1449	0.2393
RENROT	4.1825	0.0032	3.5647	0.6178	2.5643	0.5487	5.6927	6.5718
VIZYAH	3.5669	0.0395	3.1711	0.3958	/a	/a	8.3538	/a
QUMWAZ	3.7183	0.1220	3.1133	0.6050	1.9867	0.5954	0.9713	1.1714
DUXKIT	3.7703	0.0051	3.0351	0.7352	2.0066	0.4852	5.3270	0.3011
LOKQUA	3.8998	0.0605	3.1080	0.7918	2.1688	0.4969	0.2668	0.3330

a Missing data due to non-convergence of the triplet-state optimization.

## Conclusions

In summary, we developed a comprehensive computational framework for the discovery and screening of new RTP materials based on an available candidate's pool. A dataset of 218 known RTP materials was built from the literature, and the DFT and TDDFT computational results for *E*_S_1__ and *E*_T_1__ both demonstrated strong correlation with experimental values, confirming the reliability of the chosen computational methodology as a foundation for downstream screening. While several physical descriptors related to phosphorescence quantum yield were systematically evaluated, they were found to be insufficient for HTVS, motivating the aid of an ML approach. A Random Forest classification model was subsequently trained to predict two key RTP performance metrics: emission lifetime and peak wavelength. Binary classification thresholds of 90 ms for lifetime and 525 nm for emission wavelength were established, the latter criterion effectively distinguishing blue and cyan emitters from the technologically preferred green-to-NIR spectral window relevant to applications in bioimaging, anti-counterfeiting, and optoelectronics. Both classifiers achieved strong predictive performance, with F1 scores exceeding 0.84, providing a well-calibrated and practically grounded screening tool.

These models were combined into a virtual screening pipeline applied to a starting library of ∼48 000 known molecules with a small HOMO–LUMO gap from the CSD database. After the sequential application of physicochemical filters, excited-state energy criteria, and dual ML classification requiring predicted probabilities exceeding 0.75 for both lifetime and wavelength, 65 high-confidence RTP candidates were screened. Validation of their computed physical parameters confirmed their abilities and high likelihood of experimentally realizable RTP activity. Future studies will focus on experimentally validating the predictive performance of the proposed model. This work demonstrates how the principled integration of quantum chemical calculations, descriptor engineering, and ML can effectively navigate large chemical spaces to accelerate the discovery of next-generation RTP emitters.

## Author contributions

X. W. was responsible for conceptualization, methodology, investigation, formal analysis, validation, visualization, and writing the original draft of the manuscript. A. T. was responsible for supervision, project administration, and reviewing and editing the manuscript. Both authors read and approved the final manuscript.

## Conflicts of interest

There are no conflicts to declare.

## Note added after first publication

This article replaces the version published on 8th September 2026, which contained an error in eqn (4).

## Supplementary Material

SC-017-D6SC06125G-s001

## Data Availability

The databases are shared in GitHub by https://github.com/XiaWu-Liv/RTP-databases/. Supplementary information (SI) is available. See DOI: https://doi.org/10.1039/d6sc06125g.
