# Supplementary material for: Predicting room temperature phosphorescence of new organic molecules by combining physical models and data-driven methods
Source: Chem Sci. 2026 Sep 8;17(41):20088–96. doi: 10.1039/d6sc06125g (PMC13572978; doi:10.1039/d6sc06125g)
Supplement: Supplementary file 1 [file SC-017-D6SC06125G-s001.pdf]

Supplementary Information (SI) for Chemical Science.
This journal is © The Royal Society of Chemistry 2026

Supplementary data

Predicting Room Temperature Phosphorescence of New Organic Molecules by Combining Physical Models and Data-Driven Methods

Xia Wu,*[a] Alessandro Troisi *[a]

Department of Chemistry, University of Liverpool, Liverpool, L69 3BX, UK.

E-mail: xia.wu@liverpool.ac.uk, A.Troisi@liverpool.ac.uk

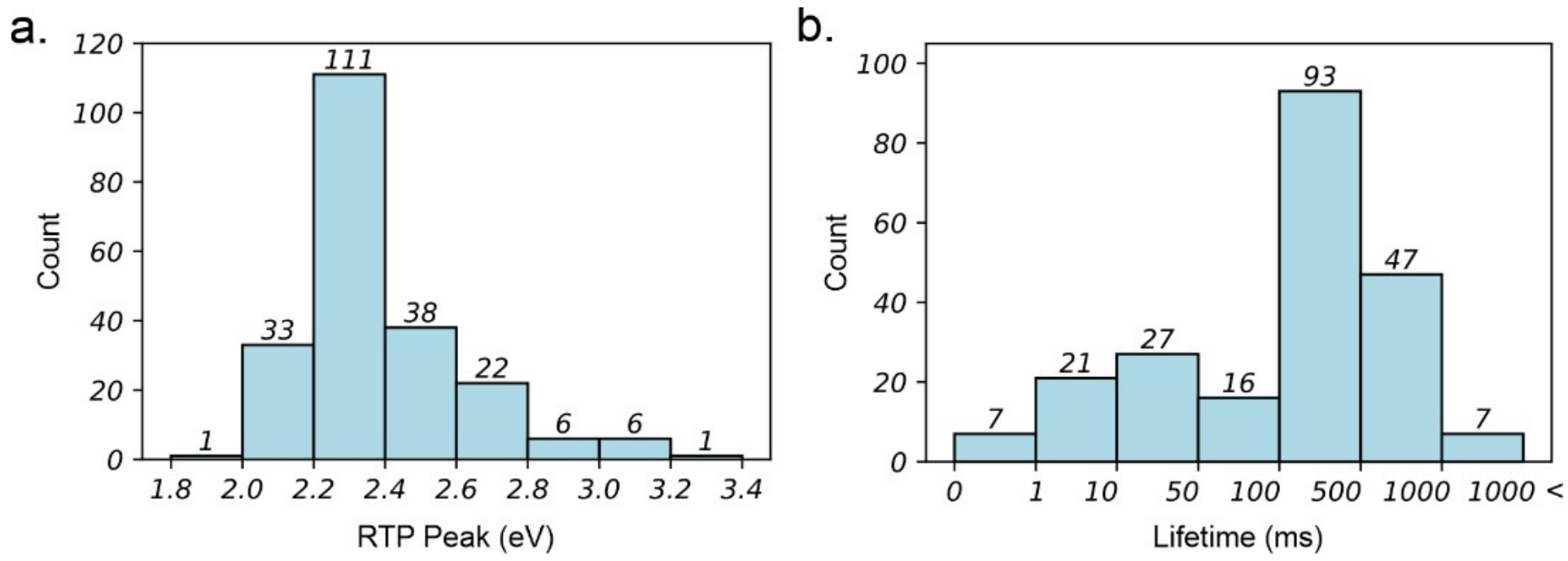

Figure S1. Distribution of the RTP wavelength and lifetime for the RTP dataset.

Table S1. Details of the descriptors using in ML model.

Descriptor	Description	Source
$E(S_1)$ (eV)	Energy of the first vertical singlet excited state	TDDFT
HOMO (eV)	Energy of the highest occupied molecular orbital	DFT
LUMO (eV)	Energy of the lowest unoccupied molecular orbital	DFT
$\Delta E(L\text{-}H)$ (eV)	Difference between LUMO and HOMO	DFT
$E(T_1)$ (eV)	Energy of the first vertical triplet excited state	TDDFT
$\Delta E(S_1T_1)$ (eV)	Difference between $E(S_1)$ and $E(T_1)$	TDDFT
$f_1(1/\Delta E_{S1T1})^2$	A descriptor related to the radiative decay rate	TDDFT
f_1	The oscillator strength of vertical S_1	TDDFT
$SUM(f_1+f_2+f_3)$	Sum of f_1, f_2, and f_3	TDDFT
D $index_{T1}$	Distance between the center of the hole and the centroid of the electron for T_1	TDDFT and Multiwfn
t $index_{T1}$	Directional separation extent of the hole and electron for T_1	TDDFT and Multiwfn
D $index_{S1}$	Distance between the center of the hole and the centroid of the electron for S_1	TDDFT and Multiwfn
t $index_{S1}$	Directional separation extent of the hole and electron for S_1	TDDFT and Multiwfn
Molecular weight	Molecular weight	RDKit
Heavy atoms	Heavy atoms	RDKit
Aromatic heavy atoms	Aromatic heavy atoms	RDKit
Fraction Csp3	Fraction Csp3	RDKit
Rotation bonds	Rotation bonds	RDKit

H-bond acceptors	H-bond acceptors	RDKit
H-bond donors	H-bond donors	RDKit
Molar refractivity	Molar refractivity	RDKit
TPSA	Topological Polar Surface Area	RDKit
WLOGP	Wildman-Crippen LogP	RDKit
Aromatic rings	Aromatic rings	RDKit

Table S2. Details of the ML model used to determine the lifetime threshold for RTP materials.

Threshold	100ms	90ms	80ms
max_depth	5	7	11
n_estimators	15	13	15
Accuracy	0.756	0.774	0.770
F1	0.834	0.846	0.844
TP/TN/FP/FN	133/31/39/14	135/33/35/14	135/32/32/18

Table S3. Details of the ML model used to determine the RTP wavelength threshold for RTP materials.

Threshold	475nm	500nm	525nm
max_depth	5	12	13
n_estimators	7	38	23
Accuracy	0.880	0.816	0.820
F1	0.934	0.885	0.859
TP/TN/FP/FN	182/9/23/3	154/23/28/12	119/59/23/16

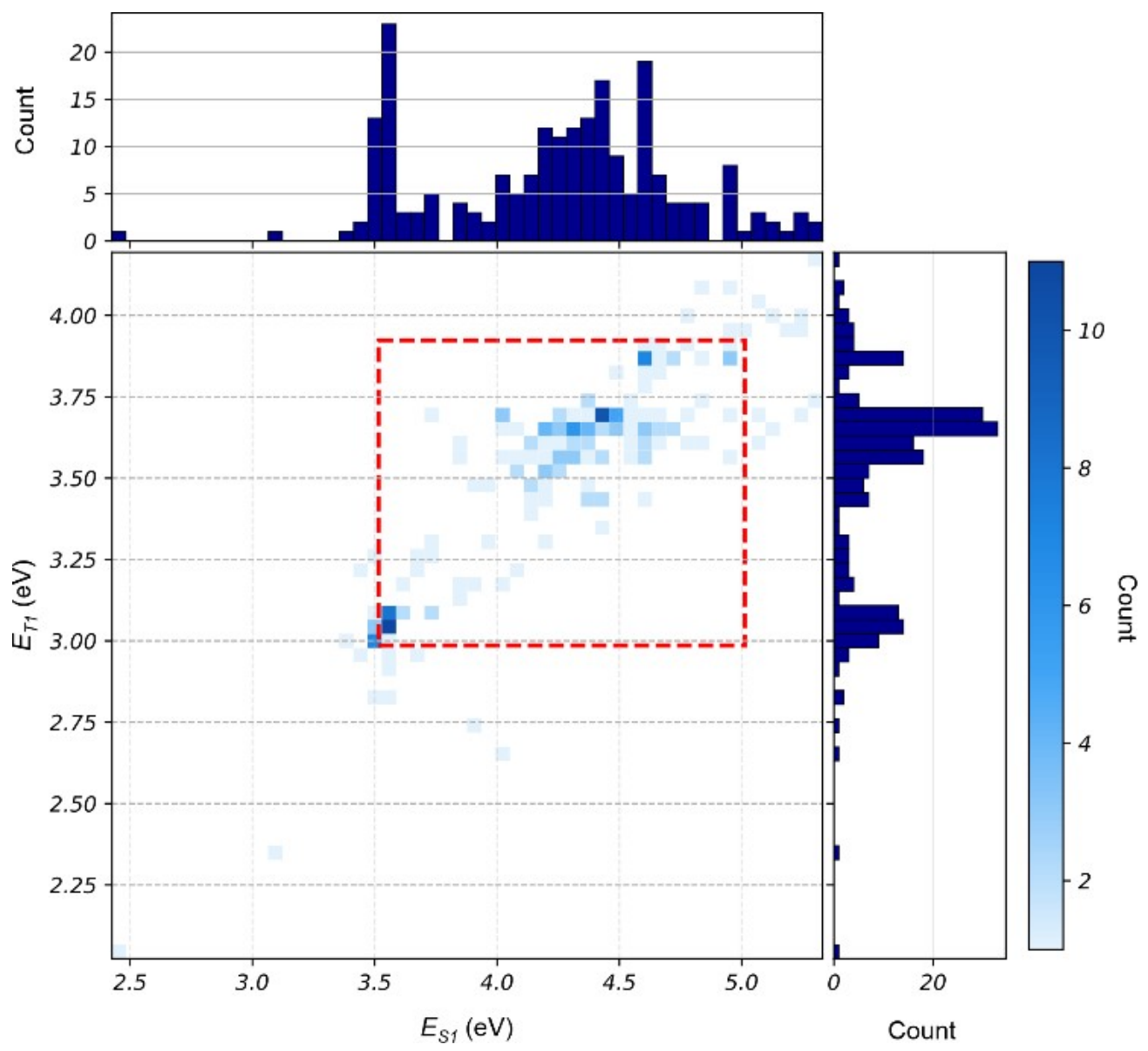

Figure S2. Distribution of E_{S1}, E_{T1} for the RTP dataset at the M06-2X/def2-SVP level. The red frame indicates the energy window containing the central 90% of the dataset for E_{S1} and E_{T1}.

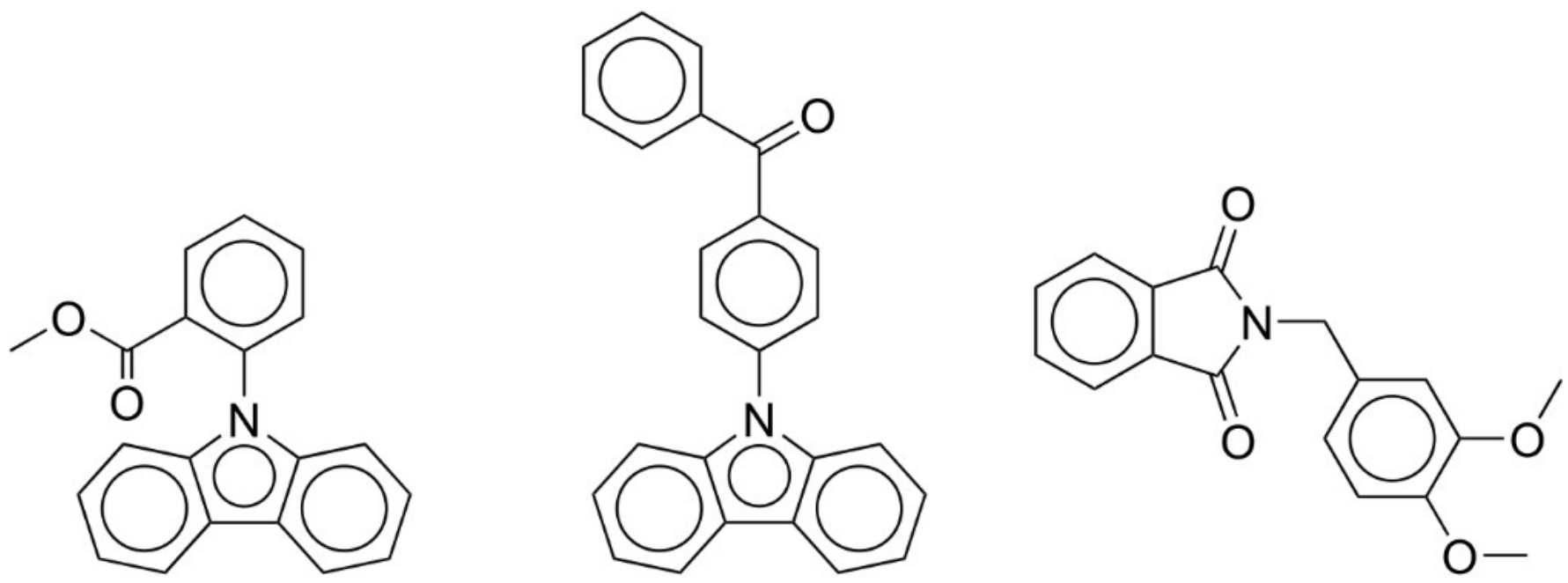

CARZIF06 FUWFAG UDIJOJ

Figure S3. Classic RTP molecules in the 65 RTP candidates reported in references.

1

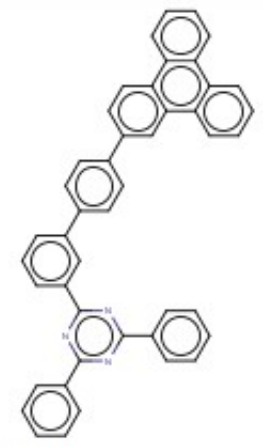

ID: RTPSOGFOM

2

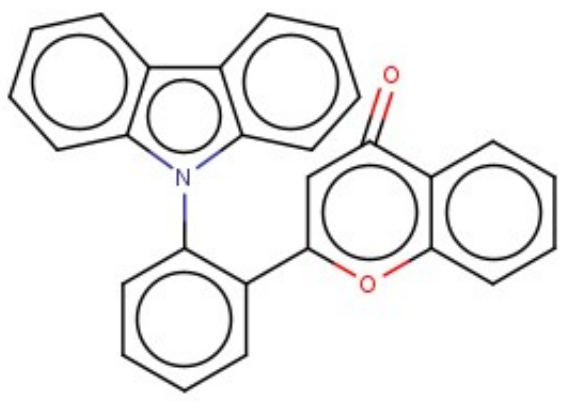

ID: RTPXIJVAQ

3

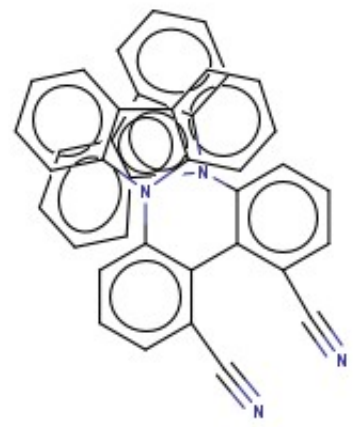

ID: RTPMUNYAY

4

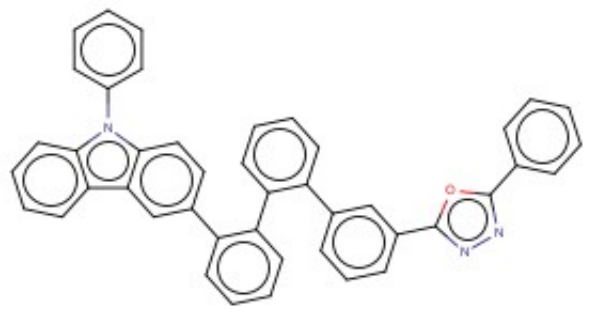

ID: RTPJAPFAK

5

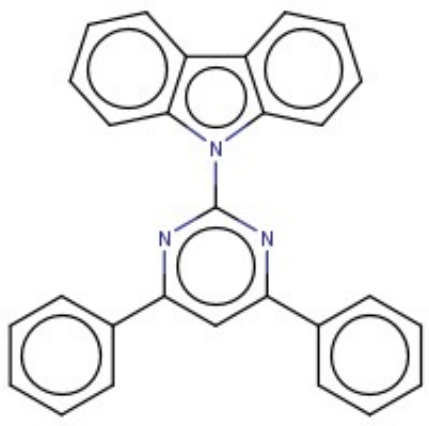

ID: RTPTUKWUU

6

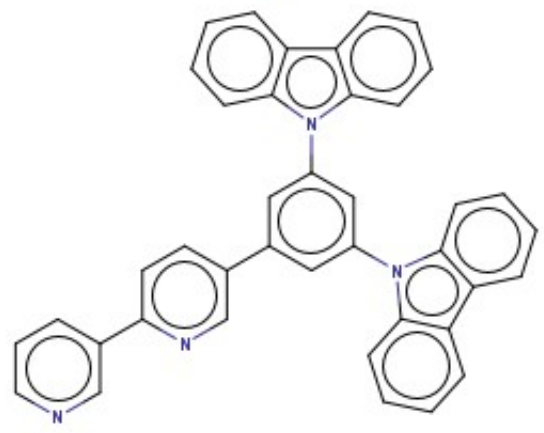

ID: RTPMIXKAI

7

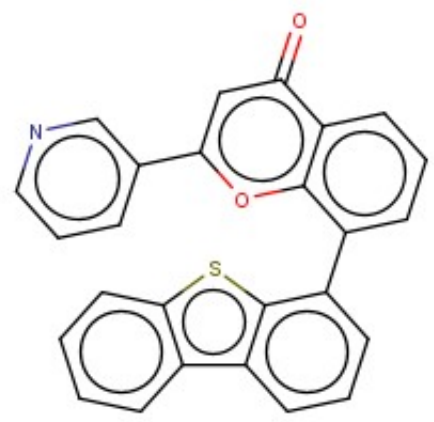

ID: RTPFUMCEY

8

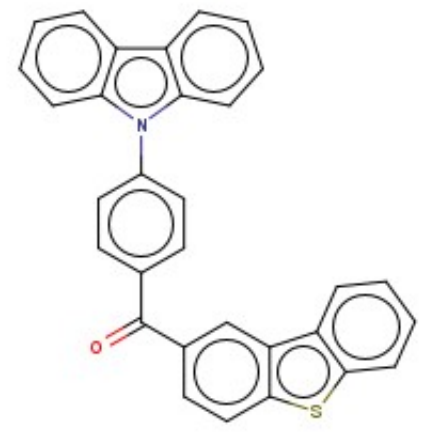

ID: RTPRUMWEE

9

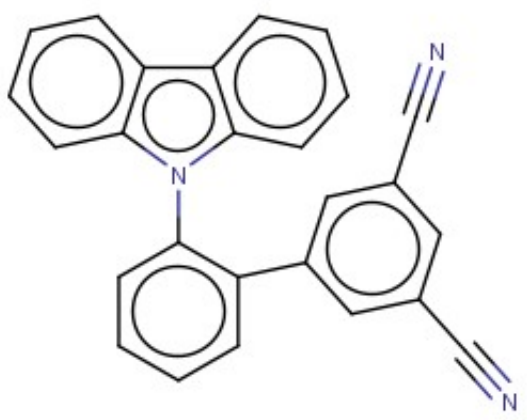

ID: RTPFONGIA

10

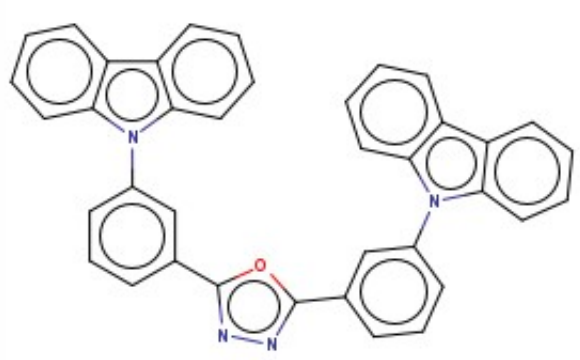

ID: RTPSUHWEY

11

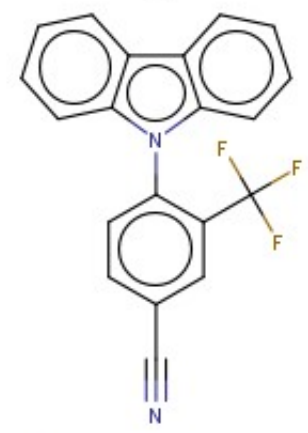

ID: RTPHIFJOY

12

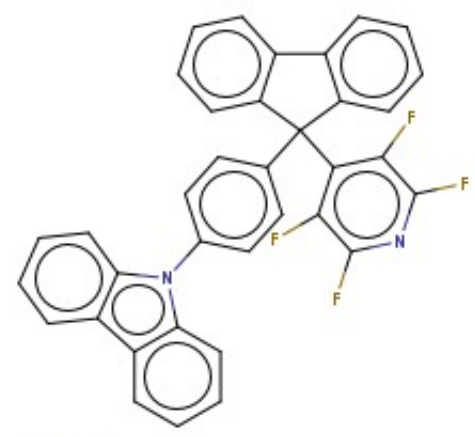

ID: RTPWEXNAR

13

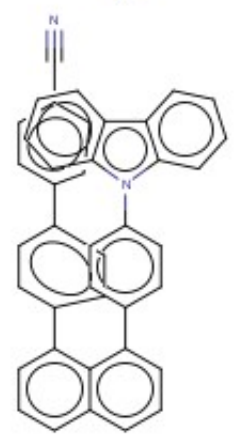

ID: RTPWENRUF

14

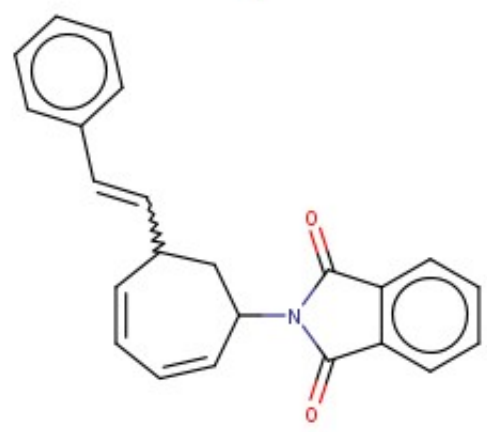

ID: RTPFEWKEZ

15

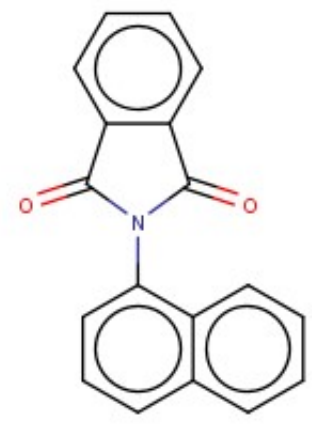

ID: RTPCEXPIF

16

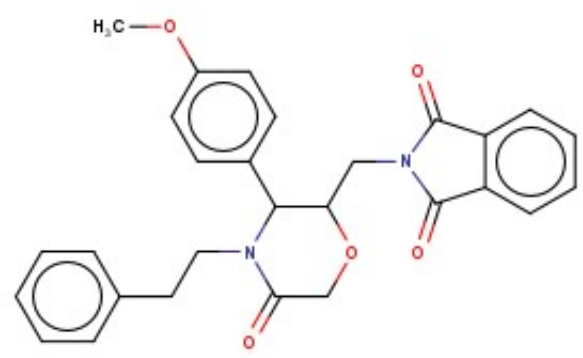

ID: RTPLOHXEN

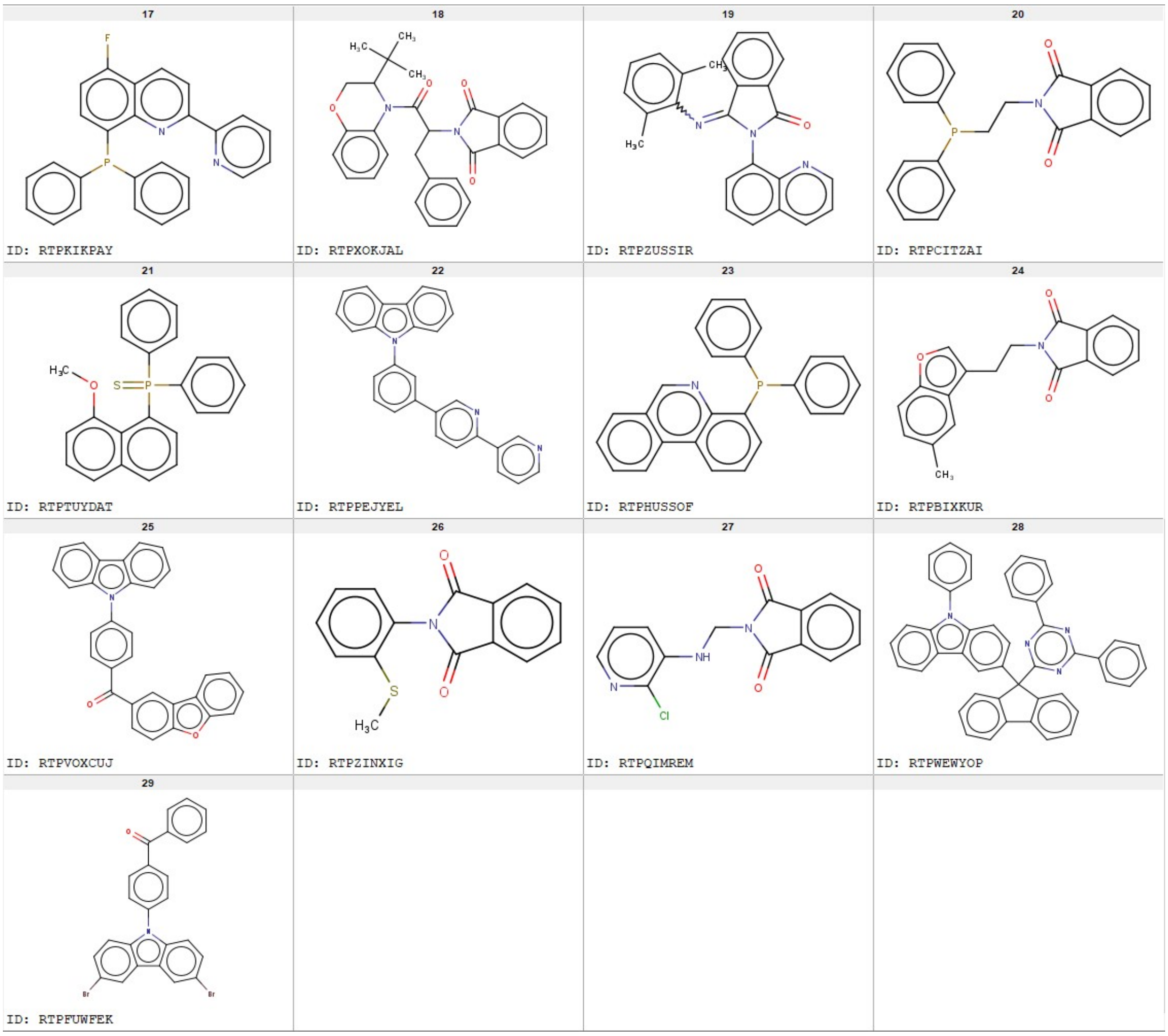

Figure S4. Molecules with similar structures as classic RTP materials in the 65 RTP candidates (computational data can be found on GitHub).

1

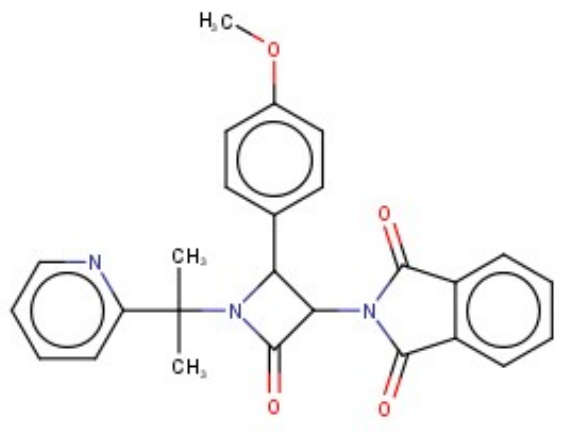

ID: RTPYIKHAD

2

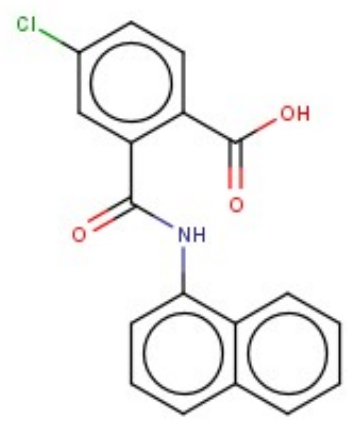

ID: RTPNPHTHA

3

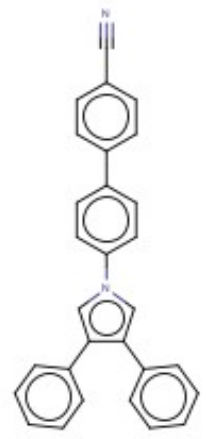

ID: RTPPIYNOD

4

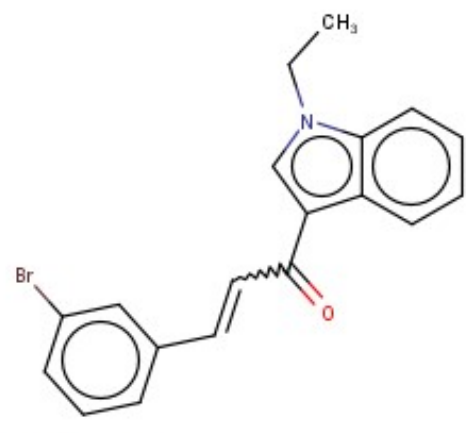

ID: RTPXUJHES

5

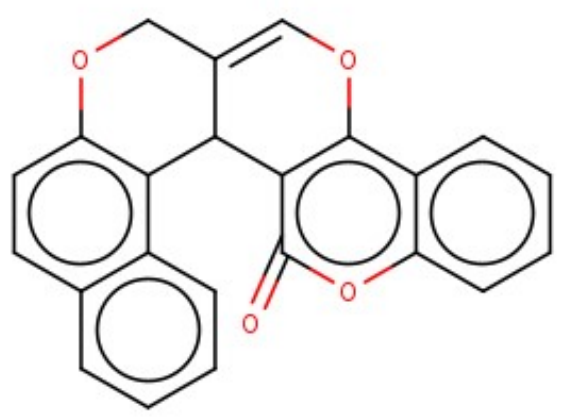

ID: RTPRUCTOZ

6

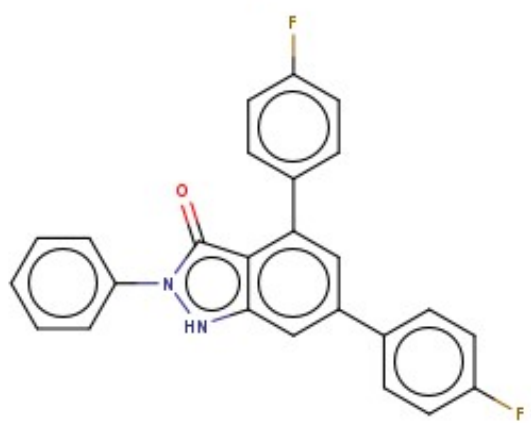

ID: RTPEROCAQ

7

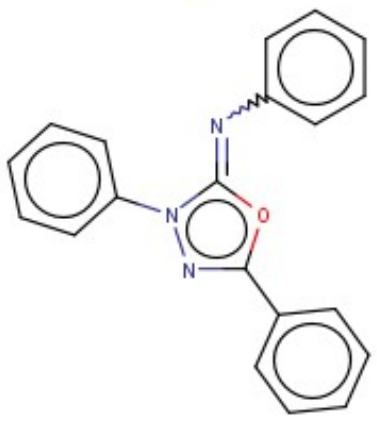

ID: RTPPIVYAX

8

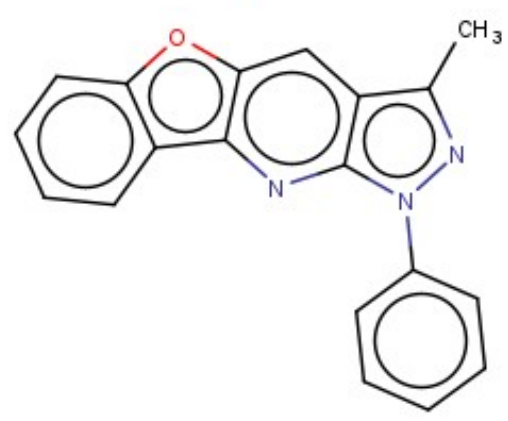

ID: RTPKOTVUM

9

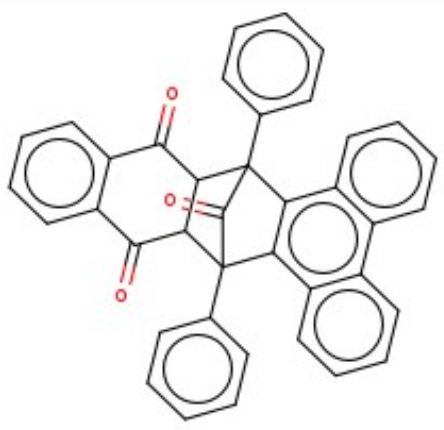

ID: RTPNAMLIX

10

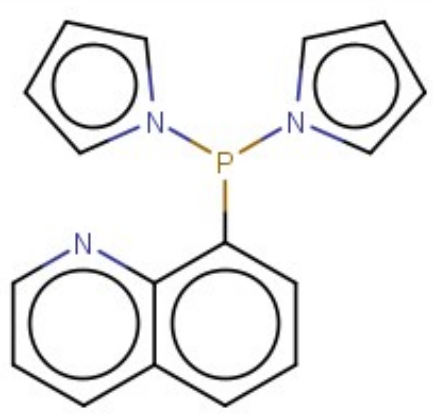

ID: RTPOCIWUT

11

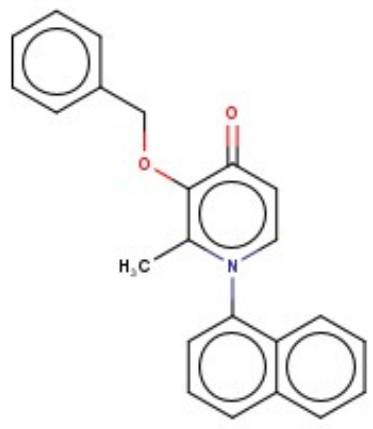

ID: RTPVACXII

12

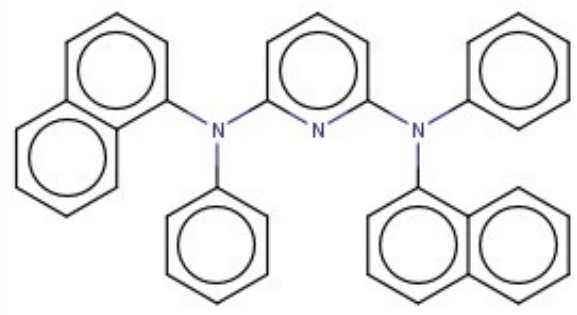

ID: RTPMERFIY

13

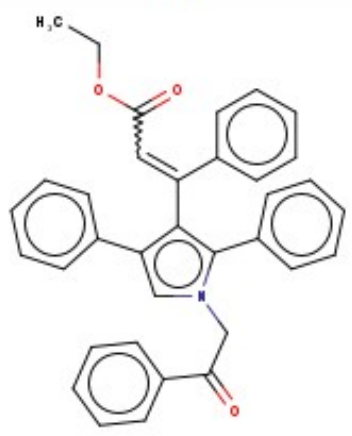

ID: RTPILOSOT

14

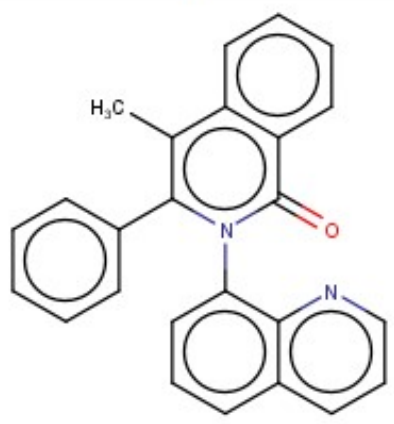

ID: RTPDIZRAH

15

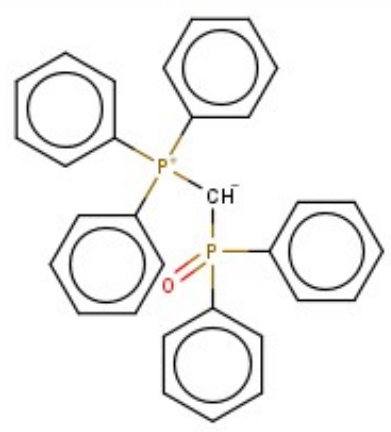

ID: RTPTAKDOZ

16

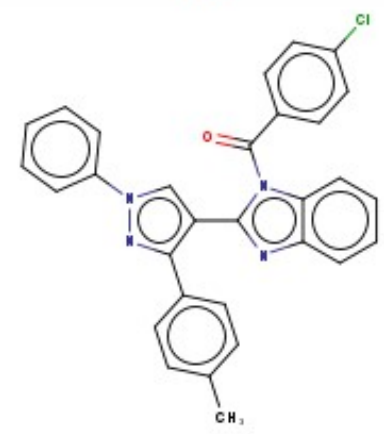

ID: RTPYIVPAX

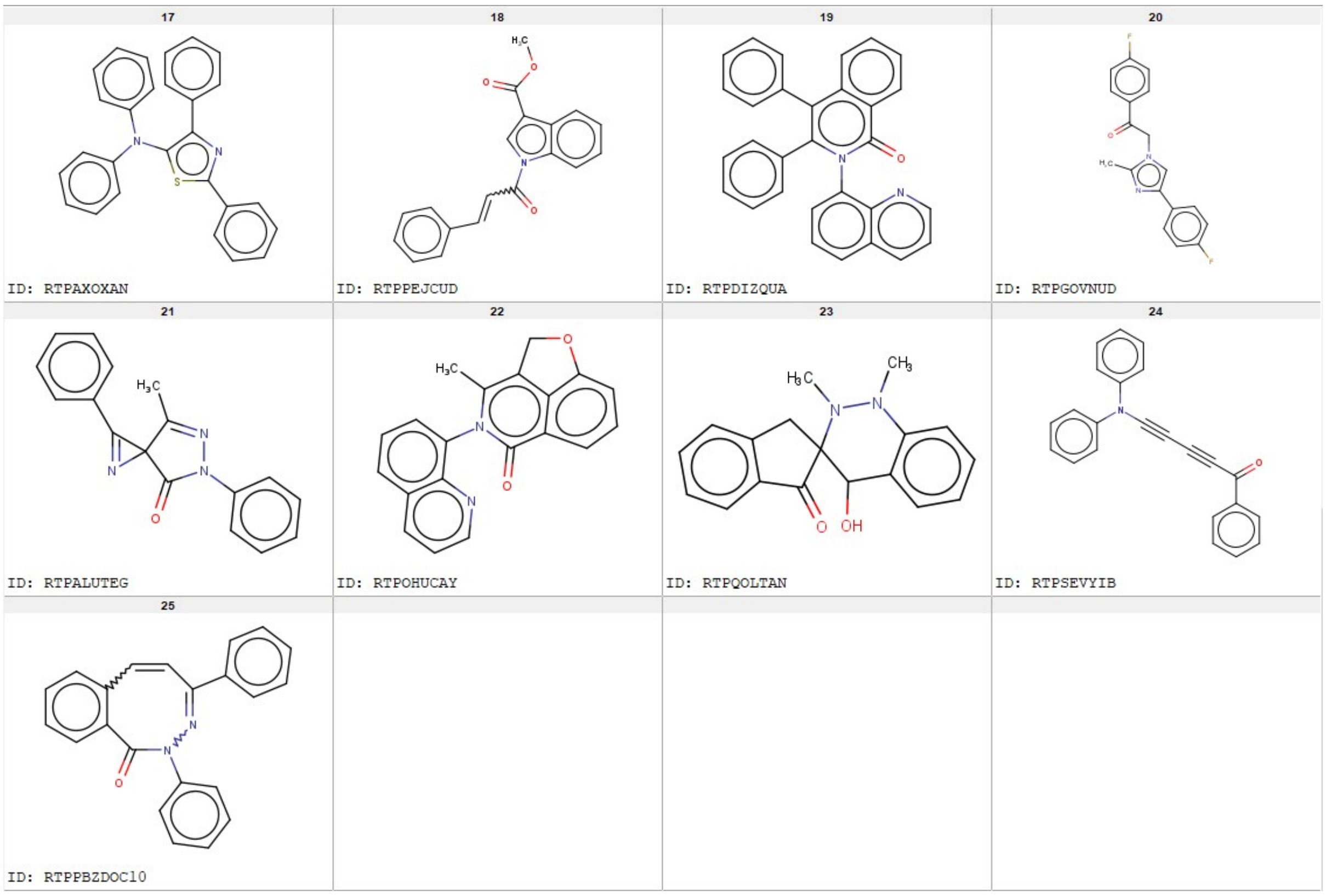

Figure S5. Part of molecular structures of promising RTP candidates featuring novel structural motifs (computational data can be found on GitHub).
